# Royal Infirmary of Edinburgh

**Published:** 1831-11

**Authors:** 


### HOSPITAL REPORTS.

### ROYAL INFIRMARY OF EDINBURGH.

### *Case of peculiar Black Infiltration of the whole Lungs, resembling Melanosis.* By JAMES CRAUFURD GREGORY, M.D., F.R.S.E., Fellow of the Royal College of Physicians, and one of the Physicians to the Royal Infirmary of Edinburgh.*

I AM induced to publish the following case, partly because I have not hitherto met with the record of any similar affection; and partly with a view of calling the attention of those practitioners who reside in the vicinity of the great coal mines, and who may have charge of the health of the miners, to the existence of a disease to which that numerous class of the community would appear to be peculiarly exposed.

John Hogg, aged fifty-nine, was admitted into the Infirmary under my care, on the 29th of March, 1831: he had gone through much service as a soldier, in America, in the West Indies, and in various parts of Europe, particularly in Spain during the Peninsular war, and had always enjoyed good health, although he had not, by his own account, been of very temperate habits, especially for the last ten or twelve years, during which period he had been employed in the coal-mines at Dalkeith.

About sixteen months before his admission, he first began to experience palpitations easily induced, with some dyspnœa and pain along the course of the sternum, which prevented him from following his occupation as a coal-miner. At the time of his admission, he complained, in addition to these symptoms, of severe cough, sometimes occurring in paroxysms, with dark-coloured viscid mucous expectoration, which had existed for five months. His breathing was rather frequent: he lay with most ease on the right side, but generally preferred the erect position; he had slight œdema of the legs, and also of the under part of the arms, near the elbows; there was some distention of the abdomen, but without fluctuation; he reported his urine to be somewhat diminished in quantity, and frequently turbid; he had never complained of pain in his loins; his bowels were habitually costive, his tongue very foul, his pulse nearly natural, and his appetite good. The impulse of the heart's action was rather stronger, and felt over a somewhat greater extent of surface, than natural, with a slightly increased sound; the respiratory murmur at that time was nowhere entirely absent, but was generally faint and bronchial, accompanied by pretty loud catarrhal râles on both sides of the chest, but chiefly, at that time, on the left side anteriorly.

As his pulse next day had risen in frequency, and was sharp and

* Edinburgh Med. and Surg. Journal.

pretty full, twelve ounces of buffy blood were drawn from the arm, and a blister was applied to the sternum, with relief to all the symptoms. On the 4th of April, the cough, expectoration, and dyspnœa had again increased, and prevented sleep; his pulse was 116, and the action of the heart still somewhat stronger than natural. The mucous and subcrepitating, or catarrhal râles were now heard generally over the left side; but the respiratory murmur was still pretty well heard under the clavicle of that side. A loud mucous râle, amounting in some points to well-marked cavernous râle, was heard under the right clavicle, and over the anterior part of that side of the chest, indicating the formation of cavities in the corresponding portion of that lung. Sixteen ounces of buffy blood were again drawn from the arm, another blister applied to the side, and afterwards leeches applied to the sternum; all with some relief: it is, however, needless to pursue the details of the treatment. He was put upon the use of various purgatives and diuretics, with some effect; but the œdema, although for some time it did not increase, never entirely disappeared. The urine continued pretty copious for some time: I examined it frequently, and, although clear, I invariably found it to become hazy on the application of heat, while its density was lower than natural, varying at different times from 1014 to 1020. From the concurrence of these two signs, and from the number of similar cases I had previously met with, I felt no hesitation in announcing that, along with the extensive disease of the lungs, and the slight organic disease of the heart, this man also laboured under a degree of that peculiar change of structure in the kidneys first described by Dr. Bright.

Early in April, the œdema extended to the scrotum and penis, and soon afterwards increased considerably in the legs and arms, while the urine diminished in quantity; the dyspnœa became more urgent, amounting at times to orthopnœa; the sputa became much more copious, and of a peculiar dark gray or nearly black colour, and his tongue acquired a coating of a similar appearance; his pulse became small, irregular in strength and frequency, and his extremities cold, notwithstanding the use of stimulants in considerable quantity. He was drowsy and slightly incoherent during the last two days, but only became comatose two hours before his death, which took place on the 18th of April.

On examination, both lungs, and particularly the right, were found to adhere strongly to the pleura costalis. The pleura pulmonalis of both lungs was much thickened; in some places, especially in the right lung, exhibiting a fibro-cartilaginous appearance and consistence, and about one fourth of an inch in thickness. The pleura costalis corresponding to this portion of the pleura pulmonalis was ossified, and had caused bony union of several of the ribs.

When cut into, both lungs presented one uniform black carbonaceous colour, pervading every part of their substance. The right lung was much disorganised, and exhibited in its upper and middle lobes several large irregular cavities, communicating with one another, and traversed by numerous bands of pulmonary substance and vessels: these cavities contained a good deal of fluid, which, as well as the walls of the cavities, partook of the same black colour. A considerable portion of the pulmonary substance surrounding them was dense, hepatised, and friable. The rest of the lung was also somewhat condensed, and very œdematous. The serum, when expressed, was of the same black colour as the substance of the lung. The left lung did not appear to contain any cavities, but was condensed, and loaded with black serum. Some minute hard points could be felt in various parts of both lungs, but they did not differ at all in colour from the surrounding substance; and no distinct tubercular deposition or infiltration could be detected in those portions of the lungs which were most hepatized, even with the aid of the microscope. The texture in these parts appeared quite uniform, and the minute hard points felt in other parts rather conveyed the impression of their being merely the ends of small bronchial branches divided in making the section. The bronchial glands did not appear enlarged, but partook of the same black colour as the substance of the lungs.

There was some dilatation, along with slight hypertrophy, of the left ventricle of the heart, and consequently some enlargement of the whole organ; the aortic valves were considerably thickened and corrugated, and, although not much shortened, could not properly have performed their office; the mitral valve was also partially thickened and opaque.

Both kidneys were of a mottled colour, and lobulated externally, but not diminished in size: the natural fibrous structure of the cortical substance of both had given place, in a great measure, to the pale granular appearance characteristic of this peculiar disease of the kidneys, which was distinctly marked, and in some places had encroached considerably on the tubular portions; in other parts these appeared healthy and entire. The liver and spleen were very friable, the liver somewhat granular in appearance. The other organs were healthy, and the black colour was entirely confined to the lungs and the bronchial glands.

The question here immediately presented itself, whether this ought to be considered as a case of infiltration of the substance of the lungs by the peculiar matter of melanosis? or whether the black colour of these organs depended merely upon the habitual inhalation of a quantity of the coal dust with which the atmosphere of a coal mine must be constantly charged, and which, remaining unabsorbed, and acting as a foreign body, had led ultimately to disorganization of the pulmonary tissue? in like manner as one form of phthisis is found to be particularly prevalent among those who, by their occupations, are most exposed to the inhalation of small irritating particles, such as stone cutters, millers, and needle grinders. In support of the first opinion, it might have been urged that the lungs have been found, more frequently than perhaps any other organ of the body, the seat of true melanotic deposition, and that the principal symptoms in this case, the dyspnœa, the anasarca, and the general cachectic habit of body in a man advanced in life, without the progressive emaciation and hectic fever of true tubercular disorganization, are precisely those which have been most frequently observed in melanosis affecting the lungs.

But there are various other circumstances which, in my opinion, render the second view much more probable. 1st. I have not met with any recorded case in which the melanotic matter was disseminated uniformly throughout the whole substance of both lungs. Haller, in his Pathological Observations, states that he had met with "a terrible species of phthisis in a man, who had one of the lobes of the lungs, (by which, I presume, he means one lung,) not indeed purulent, but full of putrid matter, as black as ink, of which he likewise found some quantity in the cavity of the thorax;" and in none of the cases recorded by Bayle, Laennec, Messrs. Cullen and Carsewell, or Mr. Fawdington, was the black colour disseminated to a greater, if even to so great an extent. 2d. Melanosis is generally found in patches or masses, which are apparently encysted; and even when found infiltrated in the substance of the lungs, some traces of the deposition of black matter may generally be found in other parts of the body. 3d. The colour of the lungs and the serum which they contained was, in this case, of a much deeper black than that of the true melanotic deposition, which usually contains an admixture of red, probably derived from the blood, giving it a brown or brownish black colour. 4th. Laennec states, in accordance with the experiments of Dr. Pearson,* that he had reason to believe that the black pulmonary matter sometimes found in such abundance in the lungs, especially in elderly people, (and which he considers as quite distinct from true melanotic matter,) proceeds, in part at least, from the inhalation of the smoke of lamps and combustible substances employed in domestic purposes; and I recollect to have heard him frequently say, that he had always found this matter most abundant in the lungs of those who had been obliged, by the nature of their occupations, to work at night and by lamplight. Were I to trust to my own experience on this point, I should say that it confirms the observation of Laennec. And, lastly, this man had enjoyed good health in various climates during the best part of his life, and he himself attributed the complaint of which he died to the air of the coal mines in which he had been working for so many years.

* On the Colouring Matter of the Black Bronchial Glands, and of the Black Spots of the Lungs. (Philosophical Transactions for 1813, part ii.)

In order, if possible, to determine this point, the washings of the lungs, containing much of the black serum, were preserved for analysis. This, although rather a tedious process, was kindly undertaken by my colleague, Dr. Christison, who has obtained the following results, which appear to me to go far to confirm the view I have ventured to take of this case, and are at least sufficient to establish the fact, that this black matter is widely different in its chemical composition from that of the ordinary matter of melanosis:

"1. Concentrated nitric acid, boiled on it, did not alter the colour.

"2. Immersion in a strong solution of chlorine had also no effect.

"3. A strong solution of caustic potass, boiled on it, took up some animal matter, and filtered very slowly. The first part which passed through was opaque and black; but the last portions were of a pale yellowish-brown colour, and transparent; so that none of the black matter was dissolved. The black matter remained on the filter, and this, well washed and dried, burned like charcoal powder, without swelling up, and with scarcely any animal empyreuma, leaving a considerable pale-gray ash.

"4. A small portion of the black powder, left after the action of boiling nitric acid, was well washed, dried, and introduced into a minute glass ball, with a tube attached, which was subsequently drawn out, by means of the spirit-lamp flame, to a fine bore. On the application of a low red heat to the ball, there was disengaged, at the open end of the tube, a considerable quantity of gas, which had the odour of coal gas, and, on the approach of a light, took fire, and burned with a dense white flame. In the tube a dark yellow fluid, likewise condensed, which had very exactly the odour of impure coal-tar naphtha, and became a soft mass on cooling, of the consistence of lard: this, when compressed between layers of filtering paper, yielded an oily stain to the paper, and left a white matter, which dissolved in boiling alcohol, and separated again on cooling, in the form of minute obscure crystals.

"In the products of this experiment, it is scarcely possible not to recognize the ordinary products of the distillation of coal. A gas of the same quality was procured, and likewise a naphthous fluid, holding in solution a crystalline principle, analogous to, if not identical with, naphthalism. I need scarcely add, that the quantity of material at my disposal was too small to allow of a more extended inquiry into its nature."

A small portion of this black powder, and the lungs themselves, have been preserved.

